# Supplementary material for: The discovery of novel noncoding RNAs in 50 bacterial genomes
Source: Nucleic Acids Res. 2024 Apr 22;52(9):5152–65. doi: 10.1093/nar/gkae248 (PMC11109978; doi:10.1093/nar/gkae248)
Supplement: gkae248_Supplemental_Files [file gkae248_supplemental_files.zip › Narunsky 50 Genomes RNA Motifs Files.zip!/Publication/61-Geobacter_sp_M21/Figures/LRC-61-30-NC_012918_1_IGR_109_2838855_2839314.sto.pdf]

LRC-61-30-NC_012918_1_IGR_109_2838855_2839314.sto.r2r

5′-GAGAUGCCUCCCGCUG●AGCRAYAUAAA●GUUAAUUAAAGUYYU●G●CYGGGCGR●GAU●YAUY●RARGCGR●●UYC○○○○○○○○○○○RGYYGAACCUGCYG●A●UUACRY●●YU●YYRG●●AR○●G●●G●G●R●●●●GC●YYY○●●G●RAACCGCUYUG●CGY●UGCYG●ARUR●UGGYCG●UU●UCAUUAUC○CGGACG●ARAAAUACGYUAAUUAG●GGCAAGAAACACGCCAAC●GRUAACUA●UUGAUAUCGCAGAGG●G●●AAGGG●RCYAARC●GG●GUACUGUAAGGGAUUCCGACGGRGAAUUGG●GAGYYGC●GRGUGCAG●YUCAUGAGCGAAAGUACUGCGAYUA●AGRGGGUUUU●YCAAAGRGGGAGYC●GYRGCG●●GUGUAAAGAAAAACGACAGCCYCGCCGC●UARCGR●GAR●YCCGG●RA●○○○○○○○○○
